# Comparative effects of gong’s mobilization and mobilization with movement in patients with adhesive capsulitis: a randomized clinical trial

**DOI:** 10.1038/s41598-025-88422-5

**Published:** 2025-02-04

**Authors:** Fareeha Amjad, Hasha Asghar

**Affiliations:** 1 College of Applied Medical Sciences, University of Hail, Hail, Saudi Arabia; 2 Riphah International University, Lahore, Pakistan

**Keywords:** Frozen Shoulder, Manual therapy, Physical therapy techniques, Rehabilitation, Health care, Rheumatology

## Abstract

Adhesive Capsulitis results in a progressive contraction of the Glenohumeral joint capsule limiting active and passive range of motion, leading to functional disabilities. Joint mobilization plays a key role in the physical therapy treatment of Adhesive Capsulitis. A relatively new technique, Gong’s Mobilization, has been introduced for the treatment of Adhesive Capsulitis. It is focused on the correction of positional faults through controlled dynamic motion of the Glenohumeral joint. The mainstay of this clinical trial is a comprehensive comparative evaluation of MWM with Gong’s Mobilization as it remains insufficiently explored. The objective of this clinical trial was to compare the effects of Gong’s Mobilization and Mobilization with Movement on pain, range of motion and functional disability in patients with Adhesive Capsulitis. In this triple blinded randomized clinical trial, sixty patients of Adhesive Capsulitis were enrolled within group A (Gong’s Mobilization) and group B (Mobilization with Movement). The treatment protocol covered 12 treatment sessions for 4 weeks. Numeric Pain Rating Scale (NPRS), Goniometer, and Urdu version of Shoulder Pain and Disability Index (U-SPADI) were used to assess the pain, range of motion and functional status respectively. These outcome measures were assessed at baseline, after 6 treatment sessions (2 weeks) and conclusively after 12 treatment sessions (4 weeks). For data analysis, within the group differences were measured by Repeated Measure ANOVA and across the group differences were measured by independent t test. A significant difference within the mean values of baseline, week 2 and week 4 NPRS, ROM, and SPADI score was observed in both study groups (*p* < 0.001). Results of independent t test used to calculate across the group differences indicated that Gong’s Mobilization was more effective in reducing disability (SPADI)(*p* < 0.001) and improving ROM(*p* < 0.001), meanwhile both groups were equally effective in reducing NPRS scores(*p* = 0.78). Moreover, a medium to large effect size was also observed for all the outcomes, pain(d = 0.5), ROM (d = 0.5–2.7), SPADI(d = 0.5). It was concluded that Gong’s Mobilization is more effective than Movement with Mobilization. Following four weeks of treatment, it pronounced statistically significant and clinically relevant results in improving pain, ROM and functional status of patients with Adhesive Capsulitis.

*Trial Registration* Trial was registered in IRCT (Trial registration number: IRCT20190717044238N4 Trial Registration Date: 01-03-2023).

## Background

Adhesive Capsulitis (AC), commonly known as Frozen Shoulder (FS), is characterized by a slow onset of pain and a restricted range of motion (ROM) in both the active and passive movements as a result of adhesions in the Glenohumeral(GH) joint capsule. Pathophysiology of FS involves synovial inflammation followed by capsular fibrosis^1^. Duplay referred to this condition as “Periarthritis Scapulohumeral” in 1872, Codman introduced the term “Frozen Shoulder” in 1932. In several research papers that have been published over the past 70 years, multiple names have been proposed, including primary idiopathic stiff shoulder, Adhesive Capsulitis, Contracture of the Shoulder, Fibrotic Capsulitis, and Frozen Shoulder^2^. Finally, Neviaser coined the term “Adhesive Capsulitis” to describe FS as the condition that impacts the shoulder’s articular capsule and triggers inflammation of the tissues accompanied by fibrosis^3^. AC is more common among middle-aged and older population between 40 and 70 years, 2–5% of the entire population is effected worldwide^4^. In US, women to men ratio for the incidence of AC is 2: 1. The disease is found to be highly associated with Diabetes Mellitus and Parkinson’s in elderly population aged 65 with a prevalence rate of 0.35%^*5*^. In Pakistan, a developing country that lacks health infrastructure and budget, FS accounts for a prevalence rate of 38%. In diabetic patients the incidence rate is even higher i.e. 66.67%^*6*^. FS is characterized by three distinct stages. The first stage is the freezing stage, shoulder pain during rest and activity is a hallmark of this stage, patients have trouble falling asleep because of night pain with a gradual decrease in ROM. The freezing stage lasts for two to nine months. In the frozen stage, stiffness is the most common complaint among the patients, with pain being a less noticeable symptom. Patients experience difficulty in performing activities of daily living like brushing hair, dressing up and overhead activities, although, at this stage patients have lesser difficulty falling asleep with minimal night pain. Frozen stage lasts for four to twelve months. The final stage is the thawing stage, that presents with absence of pain and gradual return of normal ROM lasting from five to twenty-four months^7^. The clinical picture of AC presents the reduction of passive and active ROM due to adhesion in the GH joint capsule, pain involves the entire shoulder and sometimes radiates as well^8^. As per the recent criteria to diagnose AC, radiographic imaging is not necessary as it only helps to rule out other disease conditions such as arthritis, rotator cuff tear or labral tear^9^. FS has a clinical diagnosis which is fairly based on patient’s history and examination. A limitation in capsular pattern is also observed, firstly limiting external rotation (ER), then abduction (Abd) and lastly internal rotation (IR). Although a medical diagnosis of FS can be useful in explaining tissue pathology, it is not effective in designing a rehabilitation program. History and physical examination are the primary instruments used to diagnose FS; however, imaging tests can be utilized to rule out underlying pathology. With AC, radiographs usually show no abnormalities; nevertheless, they may reveal osseous abnormalities, like GH osteoarthritis. Numerous deficits are observed in FS, but the most common one is a global loss of both active and passive ROM, FS has traditionally been defined by ROM loss of more than 25% in at least two planes, less than 30° of ER of the affected side and passive ER loss of more than 50% when compared to the uninvolved shoulder^10^.

Multiple treatment options are available for the patients of FS. Usually, a multidisciplinary treatment approach is opted for better outcomes. The treatment regime for each patient is designed according to the stage of FS. Rehabilitation can lie anywhere between the spectrum of oral medications for pain modulation with NSAIDs and corticosteroid injections, physical therapy includes conservative treatment, exercise programs and manual therapy techniques such as joint mobilization, myofascial release and strain counter strain techniques to name a few^11^. Manipulation techniques such cervical and thoracic spinal manipulations when coupled with other physical therapy modalities also have a superior effect in FS rehabilitation^12^.Lastly manipulation under anesthesia(MUA) or arthroscopic capsular release(ACR) are adopted if conservative treatment is unable to alleviate the situation^13^.

For an effective physical therapy treatment of FS, Movement with Mobilization (MWM) has been thoroughly explored as a treatment option when combined with electrotherapy and proved to have a significant effect on pain reduction and functional disability, increase in ROM has also been observed^14^. Electrotherapy usually includes hot pack, ultrasound therapy, and short-wave diathermy. Ultrasound therapy is a deep heating modality that is frequently used to treat FS, it produces molecular vibration, which aids in the breakdown of dense collagenous tissues inside the capsule^15^. The concept of MWM was introduced by Brian Mulligan. MWM has its foundations laid on the acronym “PILL.”, P stands for Pain Free, Instant and Long-Lasting Results following a mobilization. The mainstay of MWM is the correction of positional faults and tracking problems of the joints that might be the reason for the physical barrier towards the normal physiological movement^16^. The application of the technique must re-align the joint surfaces without being pain provocative in any way and the patient must report pain reduction after the treatment^17^. Gong’s Mobilization(GM) also serves as an effective technique in the treatment of FS, it was introduced by W. Gong, the mainstay of this mobilization is to correct the positional fault of the humeral head that is often medially rotated and dragged forward due to muscular tension of Subscapularis and Pectoralis Major along with the tightness of posterior capsule^18^. GM intends to keep the humeral head in its correct position during dynamic motion of the GH joint by keeping the affected scapula in anterior to posterior position and the humeral head in posterior to anterior direction at the same time^19^. W. Gong introduced GM while assessing its effects on shoulder abduction in sitting and side-lying position. On the parallel lines, Prasanth et al., Chakravarthi et al. and Babu et al. assessed the effects of GM in patients with FS comparing it with Spencer technique, Scapular and GH mobilization and conservative treatment respectively. GM proved to be more effective in these trials in contrast with the control group^18,20,21,22,23^. MWM, as per the systemic review and meta-analysis pooling 45 RCTs MWM proved to be an effective treatment for FS^24^. Pragassame et al. studied the efficacy of limited treatment frequency of MWM on twenty-six patients of FS and found MWM to be an effective technique for improving pain and ROM^25^. Sai et al. conducted a clinical trial on sixty-six patients of diabetic frozen shoulder and found MWM and supervised exercise program to be an effective strategy for the treatment of FS^26^. Another randomized controlled trial by Ravag et al. exploring the effects of MWM and Kaltenborn technique on pain and end ROM concluded that MWM is an effective technique for the treatment of FS^27^. GM and MWM target the positional faults of the joint combining controlled movement of the involved limb, that results in pain modulation and improved ROM. Both Manual Therapy Techniques initiate presynaptic inhibition of nociceptive afferent activity through peripheral mechanoreceptor activation of apical spinal neurons, reducing pain. There is a lack of literature on the comparison of the said techniques over prolonged treatment sessions and multiple treatment outcomes such as recovery of capsular pattern(ER, Abd, IR) and functional status, apart from the sole existing study conducted by Dilip et al., comparing the effects of Mulligan’s MWM and Gongs Mobilization only assessed medial rotation and pain within a maximum time period of two weeks that too with a small sample size^28^. Additionally, treatment protocols adopted in previous studies included conventional therapy exercises, so the results obtained i.e. improvement in functional status, pain reduction and increased ROM were not purely related to mobilization.

The objective of this clinical trial was to compare the effects of GM and MWM on pain, ROM and functional status of patients. We hypothesized that GM might prove to be more effective for the management of FS in terms of pain, ROM and functional status.

## Methodology

**Fig. 1:**
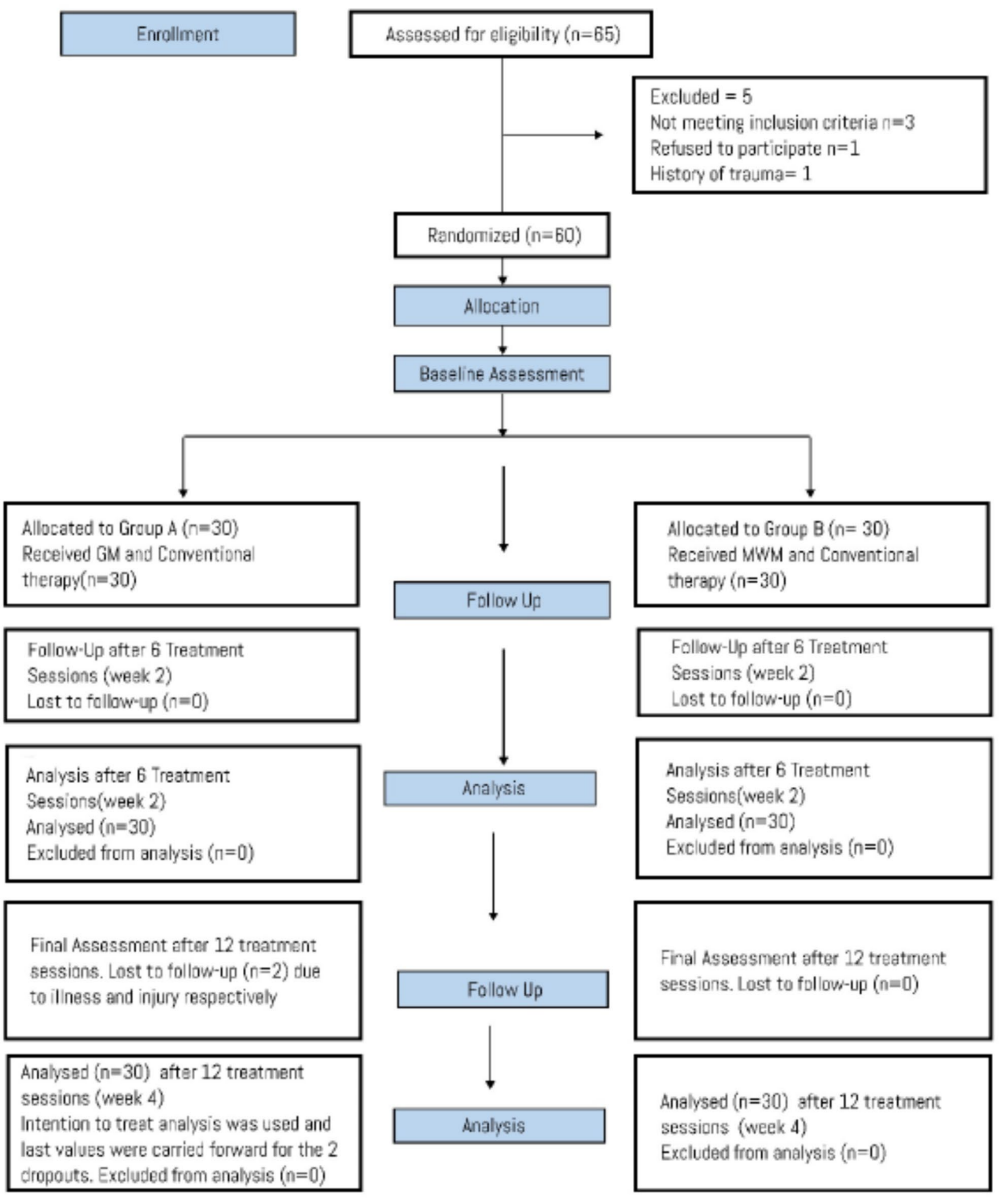
CONSORT Flow Diagram.

The sample size was calculated using online EPITOOL with 90% confidence interval and 80% power. The sample size was calculated while satisfying the NPRS values of the reference article that came out to be 54, after 10% attrition rate it was calculated to be 60(thirty participants in each group)^23^. Participants were randomly allocated through random number generator software (Research Randomizer) into two groups of 30 patients each (*n* = 30). The recruitment of patients started (2023-03-04) and the trial ended at (2023-07-03). Data was collected from the physiotherapy department of Allied Hospital Faisalabad, Punjab, Pakistan. The present clinical trial fully adheres to CONSORT guidelines^29^(Fig. 1).

### Trial design

This study was a randomized clinical trial, parallel assignment.

#### Inclusion criteria

(1) FS diagnosed at the frozen stage (progressive loss of ROM, marked stiffness with gradual pain reduction)^21,30,31^ (2) Symptoms including stiffness, shoulder pain, and limited active and passive ROM in capsular pattern (ER, Abd, IR) (3) Both male and female patients aged between 40 and 60 years with unilateral idiopathic FS (4) More than 25% painful limitation of GH ROM in two planes, less than 30° of ER of the affected side^32^.

#### Exclusion criteria

(1) Patients with history of any tumor, surgical intervention, fracture, infection, trauma and neurological deficits related to the involved shoulder complex were excluded from the study. (2) Patients using corticosteroid injections who have already been subjected to a treatment regime/exercise program for the treatment of FS during the current presentation of disease were excluded^33^. (3) Patients contraindicated to joint mobilization.(4) Patients with rheumatic diseases(Osteoarthritis, Rheumatoid Arthritis, Gout) and systemic diseases (Diabetes Mellitus and Thyroid disease) were excluded^32^.

## Procedures of the study

### Data collection tools

#### The numerical pain rating scale (NPRS)

Using NPRS, patients rated their level of discomfort on an eleven-point numeric scale as a subjective measurement. The range of the scale is 0 (no pain at all) to 10 (the greatest pain conceivable). The respondents were asked to choose the segmentation scale number that best characterized their level of overall pain. The NPRS has a moderate test-retest reliability in patients with a primary complaint of shoulder pain with ICC = 0.74, MCID of 1.1points and MDC of 2.5 points^35^.

#### Shoulder pain and disability index urdu version (SPADI)

U-SPADI was administrated to assess shoulder discomfort and impairment in an out-patient context. A five item sub-scale for pain and an eight item sub-scale for disability makes up the thirteen items in total. In a number of patient demographics, U-SPADI has ICC value of 0.89. Cronbach’s alpha is 0.94 that advocates for a higher level of internal consistency. Content Validity for each item was > 0.85^*36*^.

### Universal goniometer

AROM and PROM was measured using a universal goniometer having a good reliability for AROM with ICC ranging from 0.53 to 0.65, and SEM calculated to be 14–25°^37^. For PROM ICC ranged from 0.87 to 0.99 ^*38*^. AROM was recorded for the purpose of data analysis, Abd ROM was measured in supine position with the patient’s arm by the side. The examiner asked the patient to actively abduct the shoulder until end range. ROM was measured by placing the axis of the goniometer over head of the humerus, along with that the stationary arm was aligned parallel to the midline of the sternum and the movable arm of the goniometer was aligned with the midshaft of the humerus. For measuring ER ROM, the patient was positioned in supine and shoulder was adducted with the upper arm comfortably by the side and the elbow flexed to 90°. The examiner asked the patient to actively externally rotate the GH joint until end range. ROM was measured by placing the axis of the goniometer on the olecranon process with the stationary arm aligned with the vertical position and the movable arm aligned with the ulnar styloid process. IR was also measured in supine position, with the shoulder abducted to 90°, and the elbow placed in flexion. (In case of Abd < 90° a 45° Abd angle was employed). The examiner asked the patient to actively perform IR until the end range, placement of the goniometer was same as the one used for the measurement of ER^10^.

### Criteria for discontinuing or modifying allocated interventions for a given trial participant

- Clinically visible side effects such as increase in pain after the application of technique.
- Decrease in functional status.
- Decreased ROM due to increased pain after mobilization.

### Allocation concealment mechanism

Eligible participants were randomized into two equal groups using the random number generator (Research Randomizer) available online. Sealed opaque envelopes were used to communicate the results generated from the software and were labeled as 0 for group A and 1 for group B by the physical therapy technician who guided the patients to their assigned therapist for their particular treatment.

### Implementation

Certified and experienced orthopedic manual therapist performed the intervention techniques under the supervision of Head of Department and on the parallel lines the outcome measures were being communicated to the administration throughout the treatment period, to check for any adverse effects. No patients were harmed during the treatment period.

### Blinding

The trial was triple blinded.

- Trial participants were blinded to the group they were allocated to.
- The outcome assessor was blinded to the group patients were allocated to, the therapist giving the intervention was blinded to the aim of the study.
- Data Analyst was blinded to the clinical effects and efficacy of the treatment, as well as regarding the techniques applied to Group A and Group B.

### Intervention

Participants in Group A were treated with GM and participants in Group B were treated with MWM. Conservative treatment was ultrasound therapy for 10 min in sitting position with a pulse ratio of 1:4 and an intensity of 1.5 W/cm2 that was administrated to all the patients before mobilization^30^, along with that Codman Pendulum Exercise was prescribed as home plan as well^21,30,31,39^. Treatment protocol covered four weeks in twelve treatment sessions. Outcomes were assessed at baseline (Table 1), after the second week, at the end of the fourth week i.e. after twelve treatment sessions (Tables 2 3).

### Gong’s mobilization

**Fig. 2:**
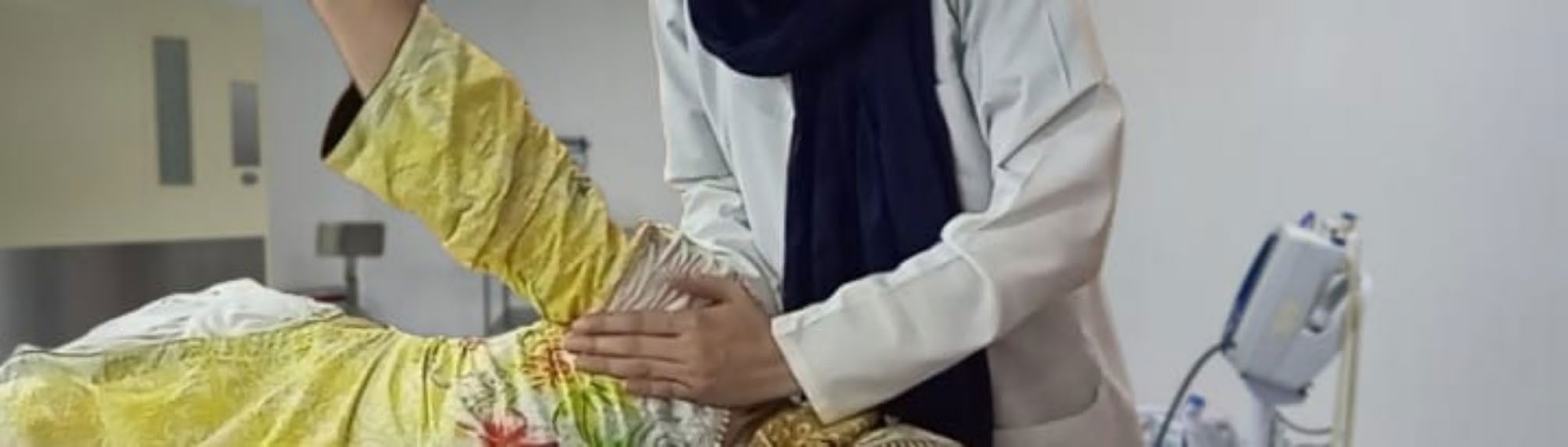
Gongs Mobilization in side-lying position.

GM was performed in side-lying position. The therapist stood on the effected side of the patient and pushed the head of humerus of the affected side in anterior to posterior direction parallel to the joint plane with the palm of his hand, simultaneously, with the other hand, the therapist pushed the scapula from posterior to anterior direction realigning and stabilizing the humeral head within correct position. The patient was then asked to quickly and powerfully perform abduction while keeping the elbow straight. Meanwhile, the therapist kept pressing the humeral head along the long axis of the humerus, maintaining the oscillatory glide in Maitland’s grade (III, IV), following the movement of the patient’s shoulder during abduction (Fig. 2). The speed of the movement was kept constant by the therapist while maintaining a little distraction at GH joint. Therapist accelerated the movement at the end range to gain GH ROM. The technique was administrated in two sets of 5 repetitions each, with 5 min of rest period between the two sets and was applied for twelve sessions^20^.

### Mobilization with movement

A positive PILL response (Pain free, Instant, Long lasting effect) was used as an assessment measure for each patient who underwent MWM.

### For shoulder abduction

**Fig. 3:**
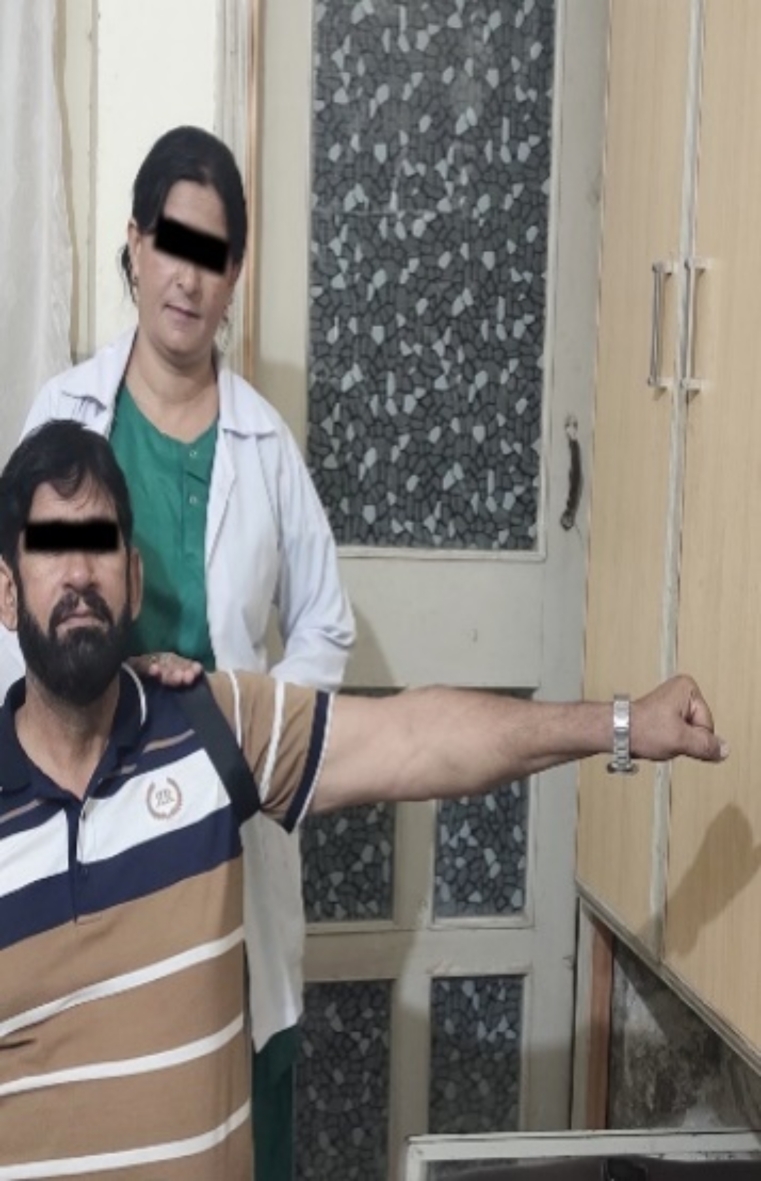
MWM for Abduction.

MWM was performed in sitting position. Therapist stood postero-lateral to the affected side placing one hand at the superior proximal humeral head of the involved shoulder and applied a lateral and inferior glide at the humeral head with the help of the mobilization belt, while patient actively performed shoulder abduction, over pressure was applied at the end range. The glide was maintained throughout the movement until the patient’s arm returned to neutral position. The procedure remained pain free for the patient throughout the session and was non-graded as per the concept of Mulligan (Fig. 3). The procedure was performed in one set of 5 repetitions and was applied for twelve sessions^40^.

### MWM for external rotation and internal rotation

**Fig. 4:**
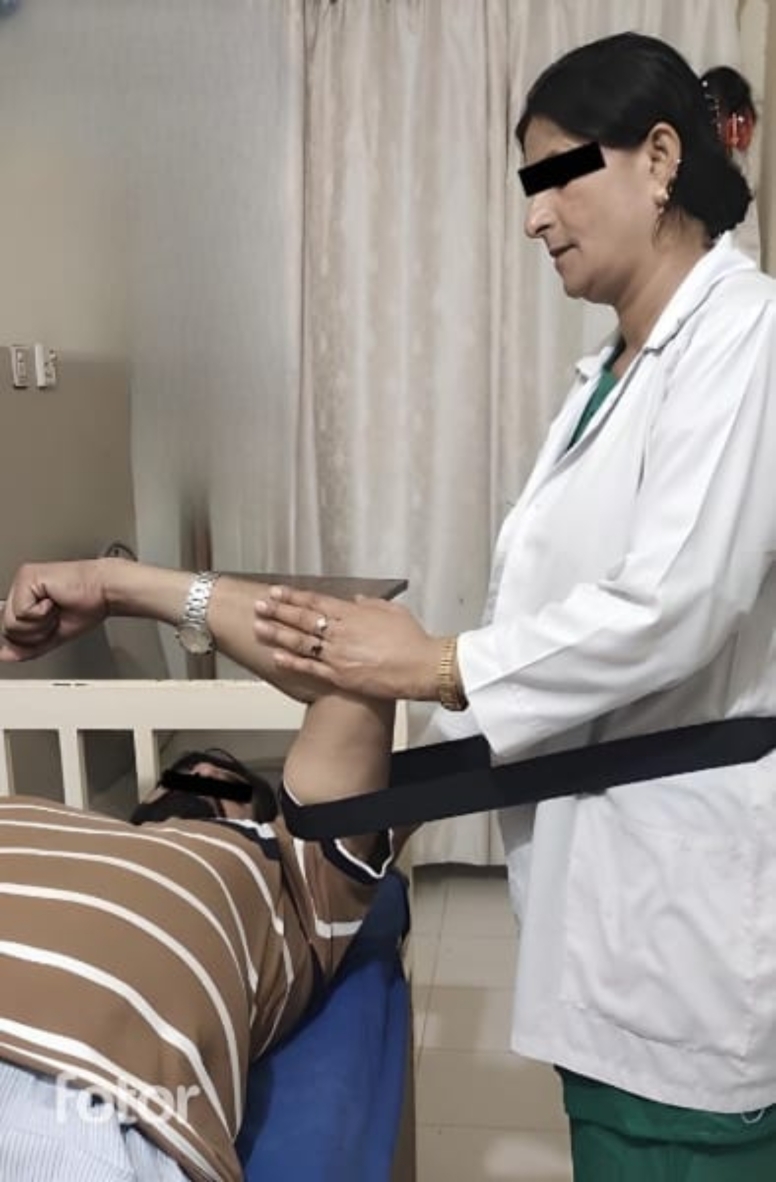
MWM for Internal Rotation & External Rotation.

The patient was lying supine with his scapula at the edge of the treatment table, the therapist stood lateral to the affected shoulder with the mobilization belt tied around the waist. The patient’s shoulder and elbow were placed at 90° flexion. Therapist grasped the distal humerus with both hands. The belt was close to the joint line, parallel to the floor. The therapist distracted the shoulder laterally and asked the patient to perform IR and ER both actively and passively. The procedure remained pain free for the patient throughout the session and was non-graded as per the concept of Mulligan (Fig. 4). The procedure was performed in one set of 5 repetitions and was applied for twelve sessions^40^.

### Data analysis

**Fig. 5:**
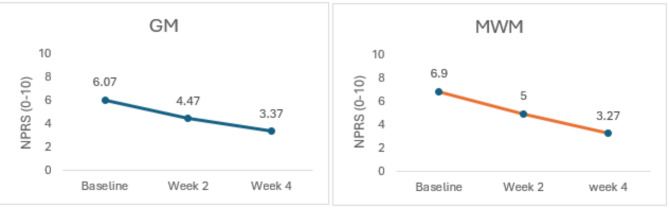
Within group comparison for NPRS at Baseline, Week 2 and Week 4.

**Fig. 6:**
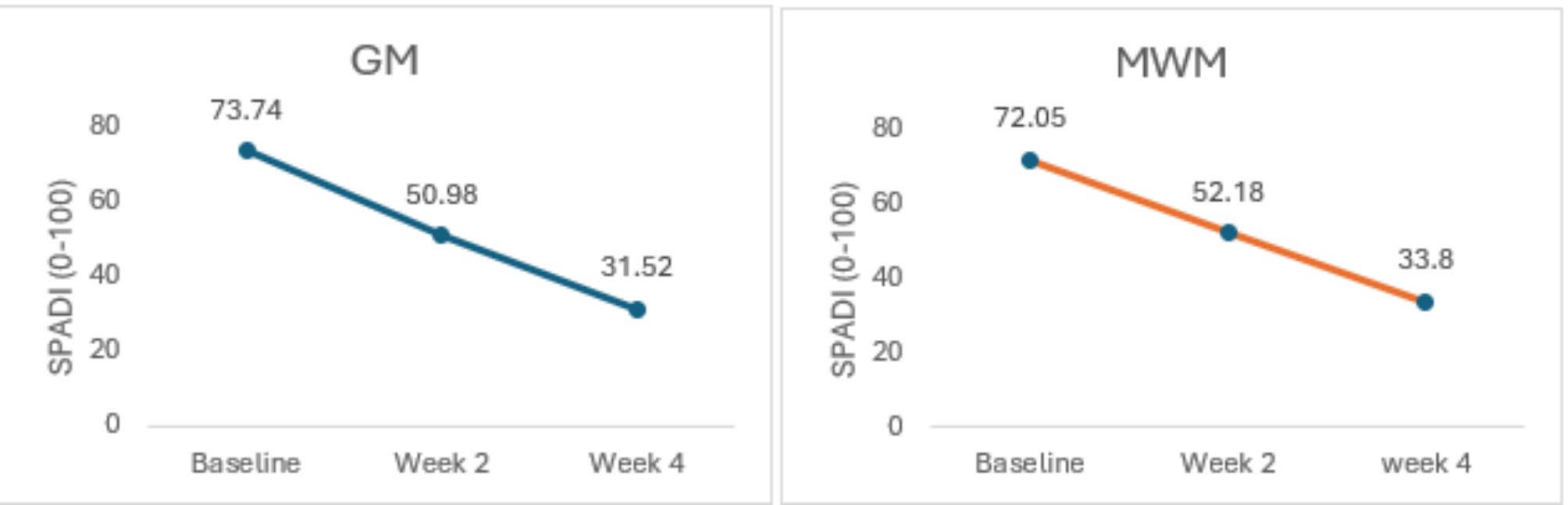
Within group comparison for SPADI at Baseline, Week 2 and Week4.

Data was analyzed by SPSS version 25. Statistical significance was set at *p* ≤ 0.05. The Kolmogorov-Smirnov test was used to check the normality of the variables. All the variables were normally distributed so parametric tests were used for calculating the Mean, Standard Deviation and Mean Difference of the outcome measures. Within the group pre and post treatment values were evaluated with repeated measure ANOVA, the outcome measures were recorded at baseline, at week 2, and at week 4 (Table 2) (Fig. 5). Across the group differences were evaluated with an independent t test, Cohen’s d calculator was used to determine the effect size to measure the clinical relevance of the results (Table 3) (Fig. 6).

## Results

### Baseline characteristics

Sixty patients(*n* = 60) with a mean age of 47.23 ± 5.77 years were enrolled in this clinical trial. The mean age of population in GM group was 47.90 ± 5.99 and within MWM group it was 46.56 ± 5.57. Mean weight of the population was 80.5 ± 9.97. No significant difference between the baseline characteristics of the two study groups (GM and MWM) in terms of NPRS(*p* = 0.07), ROM(*p* = 0.07,0.41,0.45) and SPADI(*p* = 0.09) was observed (Table 1).

#### Within the group comparison

The outcome measures at different time points are recorded in Table 2. For within group comparison Repeated Measure ANOVA was implied which revealed that, both techniques i.e. GM and MWM were effective for alleviating pain and improving ROM along with the functional status (SPADI score) of the patients with AC (*p* < 0.05). “Intention to Treat”, analysis was used for the two participants who were lost to follow-up in group A, for that purpose, last follow-up values were carried forward for data analysis.

##### After Week 2

**Fig. 7:**
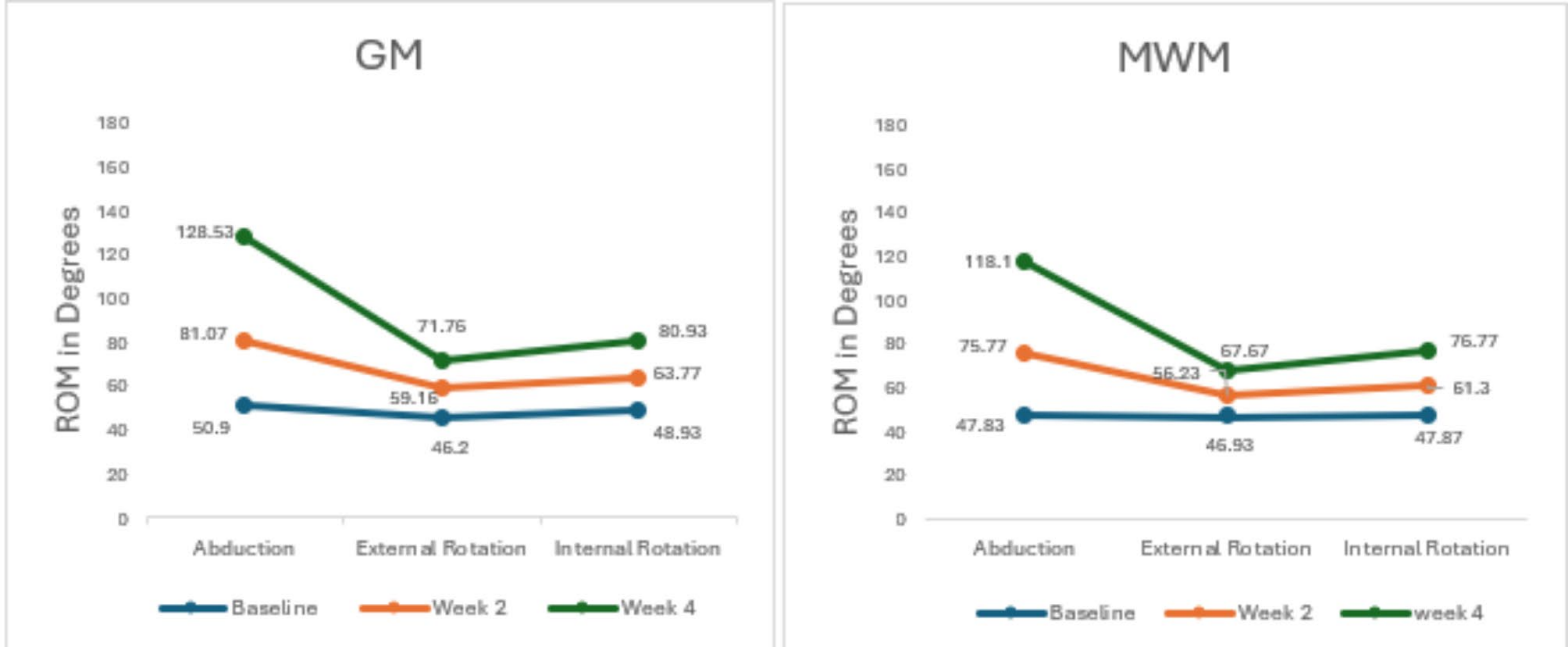
Within group comparison for ROM at Baseline, Week 2 and Week 4.

All the outcome measures showed significant improvement after week two that is evident from the results of Repeated Measure ANOVA (*p* = 0.01 − 0.001). At week two NPRS values favored GM group based on mean difference of the two groups (4.47 ± 1.47 vs. 5.00 ± 1.70)(Fig. 5). A reduction in SPADI scores that is the indicator of improved functional status also favored GM group over MWM group (50.98 ± 2.12 vs. 52.18 ± 3.08)(Fig. 6). ROM values were also inclined towards GM, Abd (81.07 ± 6.33 vs. 75.77 ± 7.36) ER (59.16 ± 3.85 vs. 56.23 ± 3.15) IR (63.77 ± 2.48 vs. 61.30 ± 3.67) (Fig. 7) (Table 2).

##### After Week 4

After twelve treatment sessions (week 4), NPRS values for both groups were almost comparable (3.37 ± 1.40 vs. 3.27 ± 1.43) (*p* = 0.01)(Fig. 5). A reduction in SPADI scores favored GM over MWM group (31.52 ± 2.18vs33.80 ± 2.26) (*p* = 0.001)(Fig. 6). ROM values were also inclined towards GM, Abd(128.53 ± 7.74vs118.1 ± 6.76)(*p* = 0.001),ER(71.76 ± 4.31vs67.67 ± 4.21)(*p* = 0.001),and IR(80.93 ± 3.05vs76.77 ± 4.36) (*p* = 0.001)(Fig. 7). The mean difference between baseline and week 4 values of all the variables was inclined towards GM (Table 2).

#### Across the group comparison

Across the group comparison was analyzed using Independent Sample t Test, for this purpose the outcome measures at different time points are recorded in Table 3. “Intention to Treat”, analysis (last follow up values were carried forward for data analysis) was used for the two participants who were lost to follow-up in group A.

##### After Week 2

**Fig. 8:**
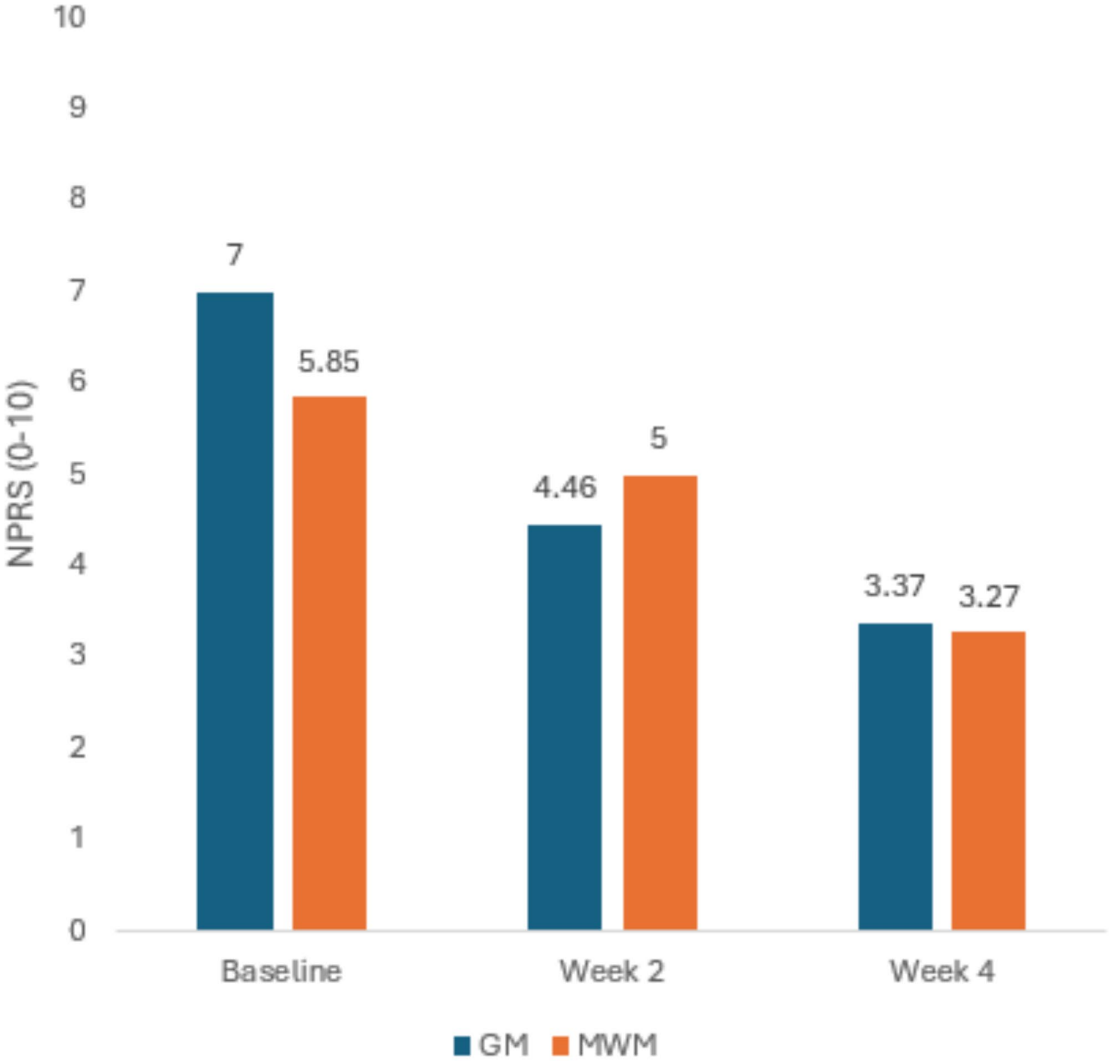
Across the group comparison for NPRS at Baseline, Week 2 and Week 4.

**Fig. 9:**
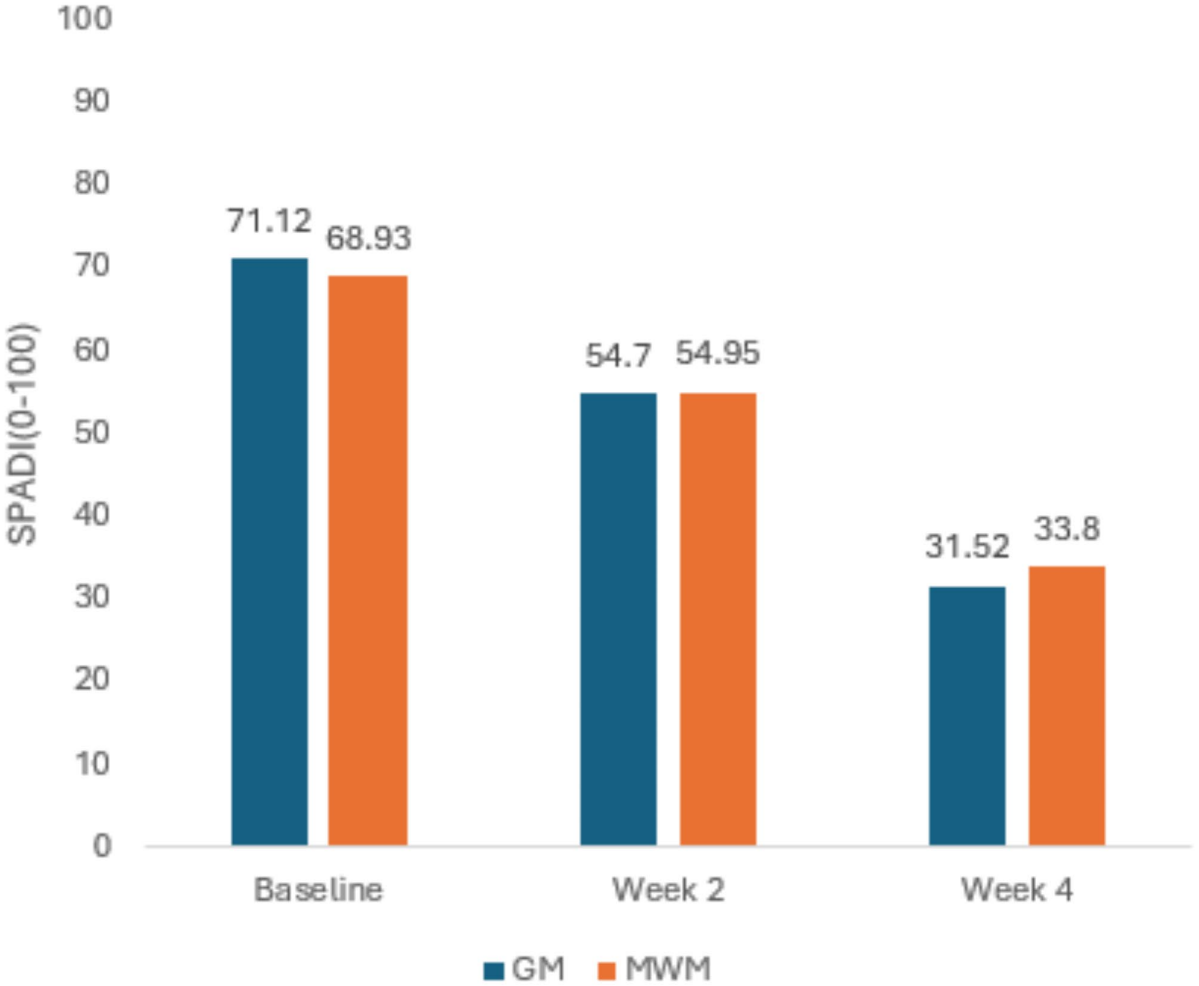
Across the group comparison for SPADI at Baseline, Week 2 and Week 4.

**Fig. 10:**
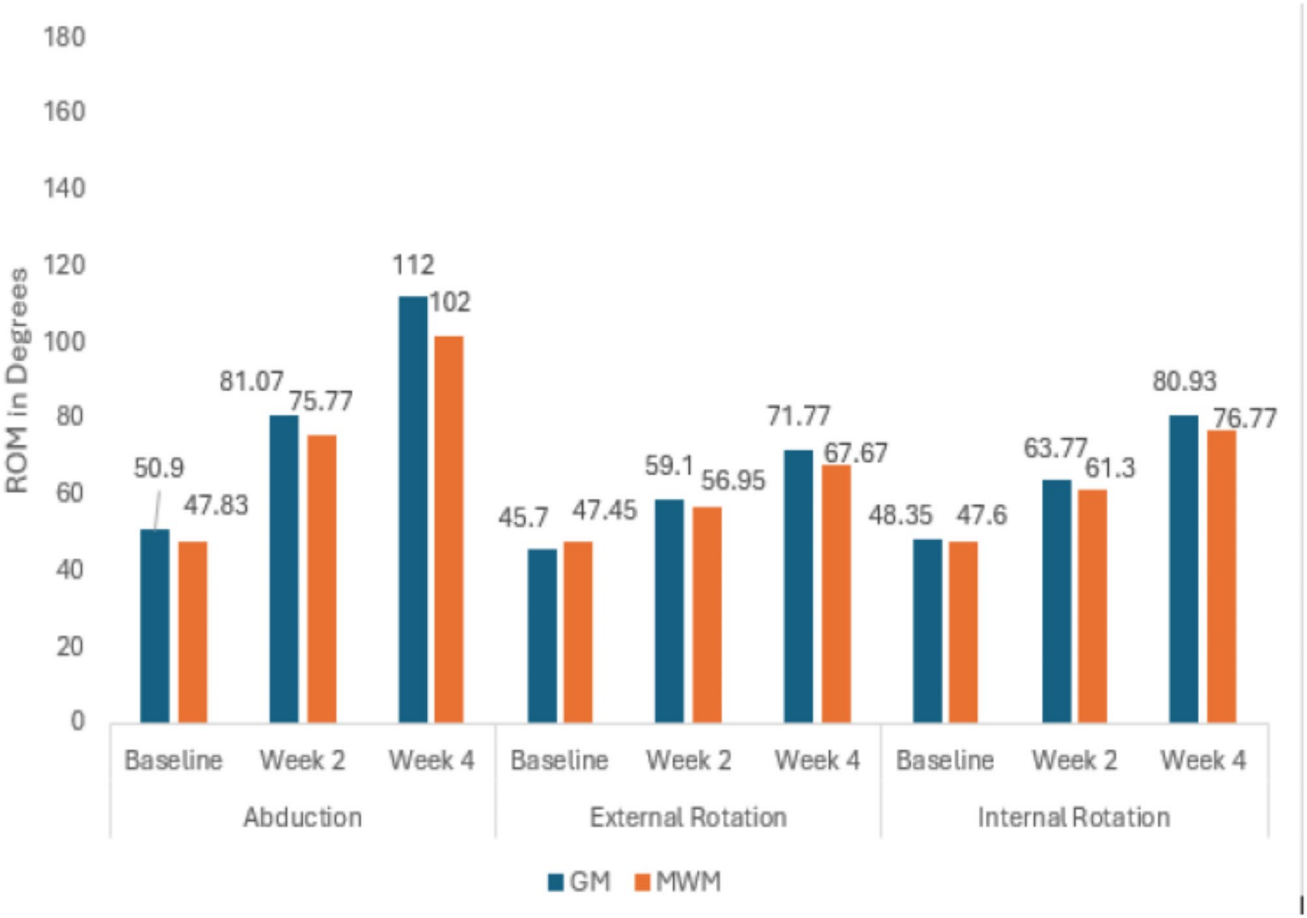
Across the group comparison for ROM (Abd, IR, ER) at Baseline, Week 2 and Week 4.

Results for Independent Sample t test after week 2 revealed that both groups GM and MWM were equally effective in reducing the NPRS score of the patients with AC (4.46 ± 1.47vs 5.00 ± 1.70) (*p* = 0.20)(Fig. 8). SPADI score also showed no significant difference at week 2 between GM and MWM group(54.70 ± 3.19vs54.95 ± 3.08) (*p* = 0.84)(Fig. 9). In case of ROM, GM was more effective in improving ER(59.10 ± 3.85vs56.95 ± 3.15)(*p* = 0.002),Abd(81.07 ± 6.72vs75.77 ± 6.30)(*p* = 0.004)and, IR(63.77 ± 2.48vs61.30 ± 3.67)(*p* = 0.004) (Fig. 10) (Table 3).

##### After Week 4

After week 4, results for Independent Sample t test indicated that, both groups GM and MWM were equally effective in reducing the NPRS score(3.37 ± 1.40vs3.27 ± 1.43) (*p* = 0.78)(Fig. 8). In case of ROM, there was a significant difference between GM and MWM group, GM group was more effective in improving ER (71.77 ± 4.31vs67.67 ± 4.21) (*p* = 0.001), Abd (112 ± 7.74vs102 ± 6.76) (*p* = 0.001) and IR (80.93 ± 3.05vs76.77 ± 4.36) (*p* = 0.001)(Fig. 10). GM also proved to be more effective for reducing SPADI score as there was significant difference between the two groups (31.52 ± 2.18 vs. 33.80 ± 2.26) (*p* = 0.001) (Fig. 9)(Table 3) .

### Effect size

Effect size was calculated using Cohens d calculator. The effect size was interpreted as (d = 0.2), medium (d = 0.5), and large (d ≥ 0.8). In terms of NPRS and SPADI a medium effect size was observed (d = 0.5). On the other hand, ROM values exhibited a medium to large effect size, ER(d = 0.5), IR(d = 2.7), Abd (d = 1.6) (Table 3).

**Table 1:** Baseline characteristics of variables.

Variables	Gongs mobilization (*n* = 30) Mean $\:\pm\:$SD	MWM (*n* = 30) Mean $\:\pm\:$SD	Total sample size(*n* = 60) Mean$\:\pm\:$ SD	*P* value
Age(Years)	47.90 ± 5.99	46.56 ± 5.57	47.23 ± 5.77	0.37
Weight(kg)	79.00 ± 9.66	82.00 ± 10.28	80.5 ± 9.97	0.24
NPRS(0–10)	6.06 ± 1.77	6.90 ± 1.78	6.48 ± 1.81	0.07
SPADI(0-100)	67.26 ± 2.63	67.34 ± 2.69	67.30 ± 2.64	0.90
Abd(Degrees)	50.90 ± 6.72	47.83 ± 6.30	49.36 ± 6.64	0.07
IR(Degrees)	48.70 ± 3.29	48.03 ± 2.97	48.36 ± 3.12	0.41
ER(Degrees)	48.10 ± 2.57	47.56 ± 3.01	47.83 ± 2.79	0.45

*NPRS: Numeric Pain Rating Scale; SPADI: Shoulder Pain and Disability Index; Abd: Abduction; IR: Internal Rotation; ER: External Rotation.

*P* < 0.05* is considered significant.

**Table 2:** Within Group comparison of Gong’s mobilization and MWM.

Outcome measures		Group	Baseline Mean$\:\pm\:$SD	Week 2 Mean$\:\pm\:$SD	Week 4 Mean$\:\pm\:$SD	Mean Difference (Week4-baseline) (95% CI)	*p* value
NPRS		GM	6.07$\:\pm\:$1.78	4.47 $\:\pm\:$ 1.47	3.37$\:\pm\:$1.40	2.70	0.01*
		MWM	6.90$\:\pm\:$1.78	5.00 $\:\pm\:$ 1.70	3.27$\:\pm\:$1.43	3.63	0.01*
Range of motion (ROM) in degrees	Abd	GM	50.90$\:\pm\:$6.72	81.07$\:\pm\:$ 6.33	128.53$\:\pm\:$7.74	61.63	0.001*
		MWM	47.83$\:\pm\:$6.30	75.77$\:\pm\:$ 7.36	118.1$\:\pm\:$6.76	54.40	0.001*
	ER	GM	46.20$\:\pm\:$2.64	59.16$\:\pm\:$ 3.85	71.76$\:\pm\:$4.31	25.56	0.001*
		MWM	46.93$\:\pm\:$3.08	56.23 $\:\pm\:$3.15	67.67$\:\pm\:$4.21	20.73	0.001*
	IR	GM	48.93$\:\pm\:$3.47	63.77 $\:\pm\:$2.48	80.93$\:\pm\:$3.05	32.00	0.001*
		MWM	47.87$\:\pm\:$3.30	61.30 $\:\pm\:$3.67	76.77$\:\pm\:$4.36	28.90	0.001*
SPADI		GM	73.74 $\:\pm\:$ 2.70	50.98 $\:\pm\:$2.12	31.52$\:\pm\:$ 2.18	42.21	0.001*
		MWM	72.05$\:\pm\:$3.07	52.18 $\:\pm\:$3.08	33.80$\:\pm\:$ 2.26	38.24	0.001*

*NPRS: Numeric Pain Rating Scale; SPADI: Shoulder Pain and Disability Index; Abd: Abduction; IR: Internal Rotation; ER: External Rotation.

*P* < 0.05* is considered significant.

**Table 3:** Across the Group Comparison of Gong’s Mobilization and MWM.

Outcome measures	Time points	GM Mean$\:\pm\:$ SD	MWM Mean $\:\pm\:$ SD	Mean difference (95% CI)	Effect size (d)	*p* value
NPRS	Baseline	7.00$\:\pm\:$1.12	5.85$\:\pm\:$1.66	$\:1.15\pm\:0.04$	0.8	0.07
	Week 2	4.46$\:\pm\:$1.47	$\:5.00\pm\:$1.70	0.54 ± 0.23	0.5	0.20
	Week 4	3.37 ± 1.40	3.27 ± 1.43	0.10$\:\pm\:$0.03	0.5	0.78
SPADI	Baseline	71.12$\:\pm\:$6.36	68.93$\:\pm\:$5.51	$\:2.19\pm\:1.12$	0.5	0.08
	Week 2	54.70$\:\pm\:$3.19	54.95$\:\pm\:$3.08	0.25 ± 0.11	0.08	0.84
	Week 4	31.52 ± 2.18	33.80 ± 2.26	$\:1.56\pm\:0.08$	0.5	*0.001
ER	Baseline	45.70$\:\pm\:$2.49	47.45$\:\pm\:$3.23	$\:1.75\pm\:$0.74	0.3	0.32
	Week 2	59.10$\:\pm\:3.85$	56.95$\:\pm\:$3.15	2.15 ± 0.7	0.5	*0.002
	Week 4	71.77$\:\pm\:4$0.31	67.67$\:\pm\:4$0.21	4.10$\:\pm\:$0.1	0.5	*0.001
Abd	Baseline	50.90$\:\pm\:6.72$	47.83$\:\pm\:6.30$	3.07$\:\pm\:$0.42	0.03	0.07
	Week 2	81.07$\:\pm\:6.72$	75.77$\:\pm\:6.30$	5.30 ± 0.42	0.5	*0.004
	Week 4	112 $\:\pm\:7.74$	102$\:\pm\:$ 6.76	10.30$\:\pm\:0.98$	1.6	*0.001
IR	Baseline	48.35$\:\pm\:$3.21	47.60$\:\pm\:$3.73	$\:0.75\pm\:$0.52	0.2	0.22
	Week 2	63.77$\:\pm\:2$0.48	61.30$\:\pm\:$3.67	2.46 ± 1.19	2.1	*0.004
	Week 4	80.93$\:\pm\:3.05$	76.77$\:\pm\:4.36$	4.16$\:\pm\:1.31$	2.7	*0.001

*NPRS: Numeric Pain Rating Scale; SPADI: Shoulder Pain and Disability Index; Abd: Abduction; IR: Internal Rotation; ER: External Rotation.

*Effect size (d); 0.2 = small, 0.5 = medium, ≥ 0.8 = large.

*P* < 0.05* is considered significant.

## Discussion

The purpose of this research was to find out how GM and MWM affected patients in frozen stage of AC. The current clinical trial assessed outcomes at three distinct stages, i.e., at baseline, after six treatment sessions, and post-treatment after twelve treatment sessions. We hypothesized that GM might be proven more effective than MWM for the treatment of FS. GM significantly reduced patients’ pain, improved ROM, and alleviated functional impairment. The findings of this study are in conformity with some of the preceding randomized clinical trials. Results demonstrated that GM and MWM are equally effective in improving pain(*p* = 0.05). This statistically significant change in NPRS values of both groups could be the result of neurophysiologic effects elicited by mobilization, stimulating the peripheral mechanoreceptors, inhibiting the type 4 nociceptors. Mobilization initiates presynaptic inhibition of nociceptive afferent activity through peripheral mechanoreceptor activation of apical spinal neurons, nonetheless mobilization results in optimal nutritional exchange at cellular level as well, hence decreasing the painful effect of stasis^14^. On the parallel lines, GM proved to be more effective in improving functional status that can be observed from the reduction of SPADI score and improved ROM of the patients affected with FS(*p* < 0.001). As per W. Gong, in GM, normal shoulder ROM occurs when humeral head is properly aligned with the articular surface, in FS humeral head is displaced in anterior-inferior direction, GM reduces tension in the posterior joint capsule by posteriorly compressing the humeral head, leading to normal rolling and sliding at GH joint. The correction of GH malalignment helps induce proper acceleration, that contributed to improved ROM in this trial^41^.

A previously conducted study comparing the effects of GM and Mulligan’s MWM by Dilip et al.^28^ only focused on two outcomes i.e. pain and medial rotation; the results indicated improvement in both study groups. However, GM group was more effective in improving ROM of the patients that is in conformity with the present clinical trial, this improvement can be attributed to the application of GM at the end range that sustains the shoulder joint in its normal position during anterior-to-posterior gliding, yielding better results for multiple treatment sessions.

Parasanth et al.^21^ also observed a significant improvement in GH ROM in GM group that is in conformity with the present clinical trial. Their study aimed to check the effects of GM and Spencer technique, recruiting thirty patients of FS. Outcome measures were assessed through NPRS, SPADI and ROM, all three outcome measures produced significant difference in the experimental group (*p* < 0.05).GM has pronounced effects on the restoration of ROM that can be a result of controlled acceleration of the effected limb at the end range activating type II mechanoreceptors which adapt and respond to capsular stretch at a rapid rate, this adaptation of mechanoreceptors to controlled dynamic motion inhibits nociceptors, which ultimately reduce NPRS and SPADI scores. A medium to large effect size for all the outcome measures was similar to the effect size calculated in the present clinical trial for each outcome (d = 0.5–0.8). GopiNath et al.,^39^ compared the effects of GM and Muscle Energy Technique on pain, ROM and functional status in patients with FS, both treatment groups received six treatment sessions. After assessing the outcome measures it was concluded that GM is more effective than Muscle Energy Technique. These results are in conformity with the present clinical trial, improvement in ROM and reduction in SPADI scores is attributable to the normal realignment of humeral head as GM is not a simple AP (anteroposterior) glide, it happens to align the articular surfaces prior to the application of repositioning force unlike any other mobilization technique. In present clinical trial, a significant reduction in SPADI score was observed within both groups GM and MWM, although, across the group comparison revealed that GM was more effective in reducing SPADI score as pre-treatment SPADI score reduced from 71.12 ± 6.36 to 31.52 ± 2.18, these results are in conformity with some of the preceding studies that produced similar results such as, clinical trial conducted by Prasanth et al.^21^ comparing GM and Spencer technique, having a treatment period in which intervention lasted five days that comprised of one session daily observed a significant reduction in SPADI score of GM group from 79.0 ± 14.17 to 31.67 ± 6.45, Gopinath et al.^39^ comparing GM with Muscle Energy Technique also observed a significant reduction in SPADI scores from 58.08 ± 9.29 to 25.28 ± 6.79. A pilot study conducted by Shrestha et al.^19^ studying the effects of GM in patients with AC produced statistically significant results with SPADI score changing from 89.97 ± 2.19 to 17.73 ± 3.21, Sah et al.^42^, while comparing the effects of GM and Cyriax manipulation also reported improved functional status in the patients of AC with SPADI score changing from 67.13 ± 4.80 to 22.30 ± 4.25 (*p* < 0.001).

Minimal Clinically Important Difference (MCID) was calculated through standard-deviation based approach that happens to be a distribution based method to calculate MCID^43^. The MCID values for NPRS in GM group and MWM were 0.56 vs. 0.83 points respectively, results were clinically meaningful for both groups i.e. as both groups showed significant reduction in NPRS scores after treatment period of four weeks. The MCID values for SPADI through standard-deviation based approach came out to be 3.18 vs. 2.75 points in GM and MWM group respectively. The MCID for ER, Abd, IR in GM group were 1.24, 3.36 and1.60 points, on the contrary for MWM group MCID values for the similar outcomes were 1.61, 3.15, and 1.9 points respectively. These values are comparable to the MCID values calculated by Inglese et al.^44^ (NPRS:0.47, IR:0.85, ER:0.83, Abd:0.57 points) and Parasanth et al.^21^ (NPRS: 0.55, SPADI:6.40, Abd: 3.52, and IR:2.40 points) who implied different approaches for the treatment of FS.

RCTs conducted by Challey et al. and Sah et al.^42,43^ comparing the effects of GM and Myofascial release technique and Cyriax manipulation respectively, concluded that GM had no superior effects on the treatment of FS, this could be a result of small sample size(*n* = 30) with a short treatment period that covered only two weeks. Ramteke et al. and Babu et al.^21^ explored the effects of GM combined with conventional physical therapy techniques in FS rehabilitation. Ramteke enrolled sixty patients in frozen stage of AC, the conventional physical therapy program included moist heating for twenty minutes, strengthening and stretching exercises and pendular exercises for two weeks. Meanwhile, the experimental group was treated with conventional therapy and GM, results of the study highlighted that GM with conventional physical therapy was more effective than conventional physical therapy alone, although functional status was not measured in this study, results were based on the outcomes of VAS and ROM only. The improvement in experimental group within 2 weeks can be the result of pairing GM with conventional therapy. Babu et al. compared the effectiveness of GM with conventional physical therapy in patients with diabetes type II who were affected with FS, the treatment period covered four weeks and ultrasound therapy for eight minutes served as baseline treatment for the both groups, conventional therapy included finger ladder exercises, Codman pendular exercises, rope and pulley exercise, shoulder towel strengthening exercise, wand exercise, and lastly, anterior shoulder stretching exercise for four weeks. The results of GM were found to be superior, patients in GM group required less treatment sessions to reduce pain, improve ROM and regain their functional status. The improvement in GM group can be attributed to GH articular repositioning offered by GM in Maitland’s grade III and IV, for the reduction of capsular tension that significantly adds to the improvement of ROM and reduces pain that is not offered by conventional physical therapy programs.

However, the present clinical trial entirely focused on the effects of mobilization, keeping ultrasound therapy and Codman’s pendulum exercise as baseline conservative treatment for both groups. So, the statistically significant results in their entirety are related to mobilization itself, either be it group A(GM) or group B(MWM). This study was a clinical trial, i.e. without a control group (that receives conventional exercises only), results could have been different if mobilizations had been paired with conventional therapy exercises and other pain-relieving modalities.

## Limitations of study

Passive ROM was not recorded to reduce the risk of bias in data collection. Based on the intent of this clinical trial, outcome measures such as pain, SPADI score, and ROM in capsular pattern were observed but changes in other parameters related to the disease such as stress levels and sleep disturbance were not recorded through related questionnaires. Patients were not very responsive in filling the self-report questionnaire, some found it lengthy and tiresome.

## Conclusion

The results of this study concluded that GM produced statistically significant and clinically relevant results for all outcome measures. GM proved to be more effective for improving ROM and functional status than MWM in patients with AC.

### Recommendations

- It is advised to conduct further research to check the long-term clinical implications of these interventions by proceeding follow-up sessions.
- Further study is advocated to check the combined effects of both techniques using a cross over or nested study design, in patients with AC as both techniques were effective for disability and pain in patients with AC.
- Although Urdu version of SPADI was used for data collection, patients were reluctant to fill the questionnaire on their own, some patients found it tiresome and lengthy, a concise version of SPADI is needed to make it a true self report questionnaire for Urdu speaking population.

#### Clinical implication

Clinical implication of this study is the application of GM along with ultrasonic therapy, it can be considered beneficial for improving ROM in AC patients because of its immediate effects.

## Data Availability

The datasets generated and analysed during the present clinical trial are not publicly available due to limitations of ethical approval involving the patient data but can be made available from the corresponding author on reasonable request.

## Abbreviations

NPRS: Numeric Pain Rating Scale
SPADI: Shoulder Pain and Disability Index
MWM: Mobilization with Movement
AC: Adhesive Capsulitis
GM: Gong’s Mobilization
